# Iron-Dependent Regulation of Hepcidin in Hjv−/− Mice: Evidence That Hemojuvelin Is Dispensable for Sensing Body Iron Levels

**DOI:** 10.1371/journal.pone.0085530

**Published:** 2014-01-07

**Authors:** Konstantinos Gkouvatsos, Carine Fillebeen, Alina Daba, John Wagner, Giada Sebastiani, Kostas Pantopoulos

**Affiliations:** 1 Lady Davis Institute for Medical Research, Jewish General Hospital, Montreal, Quebec, Canada; 2 Division of Gastroenterology, Royal Victoria Hospital, McGill University Health Center, Montreal, Quebec, Canada; 3 Department of Medicine, McGill University, Montreal, Quebec, Canada; The John Curtin School of Medical Research, Australia

## Abstract

Hemojuvelin (Hjv) is a bone morphogenetic protein (BMP) co-receptor involved in the control of systemic iron homeostasis. Functional inactivation of Hjv leads to severe iron overload in humans and mice due to marked suppression of the iron-regulatory hormone hepcidin. To investigate the role of Hjv in body iron sensing, Hjv−/− mice and isogenic wild type controls were placed on a moderately low, a standard or a high iron diet for four weeks. Hjv−/− mice developed systemic iron overload under all regimens. Transferrin (Tf) was highly saturated regardless of the dietary iron content, while liver iron deposition was proportional to it. Hepcidin mRNA expression responded to fluctuations in dietary iron intake, despite the absence of Hjv. Nevertheless, iron-dependent upregulation of hepcidin was more than an order of magnitude lower compared to that seen in wild type controls. Likewise, iron signaling via the BMP/Smad pathway was preserved but substantially attenuated. These findings suggest that Hjv is not required for sensing of body iron levels and merely functions as an enhancer for iron signaling to hepcidin.

## Introduction

Dietary iron absorption and systemic iron homeostasis are controlled by hepcidin, a peptide hormone [1], [2]. Hepcidin is secreted by the liver and targets intestinal enterocytes, reticuloendothelial macrophages and other cell types. It operates by binding to the iron transporter ferroportin on the plasma membrane of target cells, which promotes ferroportin ubiquitination, internalization and degradation in lysosomes [3]. Thereby, hepcidin inhibits iron fluxes to the bloodstream. The expression of hepcidin is induced by increased plasma or hepatic iron, inflammatory signals, or ER stress [4], [5]. Iron-regulation of hepcidin involves BMP/Smad signaling and requires the activities of HFE, transferrin receptor 2 (TfR2) and hemojuvelin (Hjv). Pathogenic mutations in either of these proteins lead to mild or severe impairment of the hepcidin pathway, and compromise its responsiveness to iron. This results in iron overload (hemochromatosis) due to enhanced absorption of dietary iron [6], [7]. The most common variant of this genetic disease is caused by mutations in the hemochromatosis protein HFE, which is associated with mild hepcidin insufficiency. Severe hepcidin insufficiency leads to early onset juvenile hemochromatosis. This is caused by genetic disruption of either the *HAMP* or the *HFE2* gene, encoding hepcidin or Hjv, respectively.

Hjv is a member of the repulsive guidance molecule family that is expressed in hepatocytes and in striated muscles [8]. Its role as an upstream regulator of hepcidin was established by genetic and biochemical studies [9]–[14]. Thus, patients with mutated, non-functional Hjv fail to appropriately upregulate hepcidin in response to iron and develop juvenile hemochromatosis [9]. A similar phenotype has been documented in mice bearing complete [10], [11] or liver-specific [12], [13] ablation of Hjv. At the biochemical level, Hjv functions as a BMP co-receptor and promotes efficient iron-dependent BMP/Smad signaling to hepcidin [14].

Hjv was proposed to be essential for dietary iron sensing [10] but the underlying mechanism has not been studied thus far. To address this issue, we employed Hjv−/− mice and analyzed their molecular responses to dietary iron challenges. We show that these mice retain a capacity for residual iron-dependent regulation of hepcidin mRNA expression, which is exclusively driven by hepatic iron stores. Nevertheless, this response is blunted due to attenuated Smad signaling. Our data suggest that Hjv is dispensable for sensing alterations in body iron levels and provide evidence that the major function of this protein is to amplify the primary iron signal.

## Materials and Methods

### Animals

All experimental procedures were approved by the Animal Care Committee of McGill University (protocol 4966). Hjv−/− mice in an inbred 129S6/SvEvTac strain [11] were kindly provided by Dr. Nancy Andrews (Duke University) and backcrossed for ten generations to the C57BL/6 genetic background. Animals were housed in macrolone cages (up to 5 mice/cage, 12∶12 h light-dark cycle: 7 am – 7 pm; 22±1°C, 60±5% humidity) according to standard institutional guidelines. Ten-week old male C57BL/6 Hjv−/− mice and isogenic wild type controls (n = 10 per each group) were placed on diets with variable iron content (Harlan Laboratories, Indianapolis) for four weeks. The standard diet contained 225 ppm iron (2018 Teklad) and the low-iron diet 75–100 ppm iron (TD.05616). The high-iron diet was the standard, enriched with 2% carbonyl iron (TD.09521). At the endpoint, the animals were sacrificed by cervical dislocation.

### Serum Biochemistry

Blood was collected with cardiac puncture. Serum was separated by centrifugation and used to determine Tf saturation, iron and ferritin concentration by a Roche Hitachi 917 Chemistry Analyzer.

### Tissue Iron Quantification

Hepatic and splenic non-heme iron content was quantified by the ferrozine assay [15]. Results are expressed as micrograms of iron per gram of dry tissue weight.

### Histological Analysis

Tissue specimens were fixed in 10% buffered formalin and embedded in paraffin. Deparaffinized sections were stained with H&E (to monitor tissue architecture) or Perls’ Prussian blue (to visualize ferric iron deposits).

### Quantitative Real-time PCR (qPCR)

Total RNA was isolated from frozen tissues using the RNeasy Mini kit (Qiagen). Purity was assessed by 260/280 nm absorbance ratios and quality was monitored by agarose gel electrophoresis. qPCR was performed as previously described [15], by using gene-specific primers (Table 1). Data were analyzed with the Pfaffl method [16] using β-actin as housekeeping gene. The qPCR results are represented as fold changes compared to wild type mouse samples, from mice fed a normal iron diet.

**Table 1:** List of primers used for qPCR or genotyping.

Gene	GenBank accession No	Forward primer sequence	Reverse primer sequence
Actb	NM_007393.3	GACGACATGGAGAAGATCTG	GTGAAGCTGTAGCCACGCTC
Bmp6	NM_007556.2	ACTCGGGATGGACTCCACGTCA	CACCATGAAGGGCTGCTTGTCG
Hamp1	NM_032541.1	AAGCAGGGCAGACATTGCGAT	CAGGATGTGGCTCTAGGCTATGT
Hfe2	NM_027126.4	ATCCCCATGTGCGCAGTTT	GCTGGTGGCCTGGACAAA
Id1	NM_010495.2	GGTACTTGGTCTGTCGGAGC	GCAGGTCCCTGATGTAGTCG
Smad7	NM_001042660.1	TCGGACAGCTCAATTCGGAC	GGTAACTGCTGCGGTTGTAA

### Western Blotting

Liver lysates were prepared as described earlier [17]. All samples (containing 30 µg of protein) were analyzed by SDS-PAGE on 10% gels by standard procedures. Following transfer of the proteins onto nitrocellulose filters (BioRad), the blots were saturated with 5% non-fat milk in phosphate buffered saline (PBS) containing 0.1% (v/v) Tween-20 (PBS-T) for 1 hour, and probed overnight with a 1∶1000 diluted antibodies against phospho-Smad1/5/8 or Smad1 (Cell Signaling). After three washes with PBS-T, the blots were incubated with 1∶25000 diluted peroxidase-coupled goat anti-rabbit IgG (Sigma) for 1 hour. The peroxidase signal was detected by enhanced chemiluminescence with the Western Lightning ECL kit (Perkin Elmer) and quantified by densitometry.

### Statistics

Statistical analysis was performed by using the GraphPad Prism software (v. 5.0d). Data from two groups were analyzed by the Student’s t test. Data from multiple groups were analyzed by one-way ANOVA with Bonferroni post-test correction. A probability value p<0.05 was considered to be statistically significant.

## Results

### Pathophysiological Responses of Hjv−/− and Wild Type Mice to Dietary Iron Manipulations

**Figure 1:**
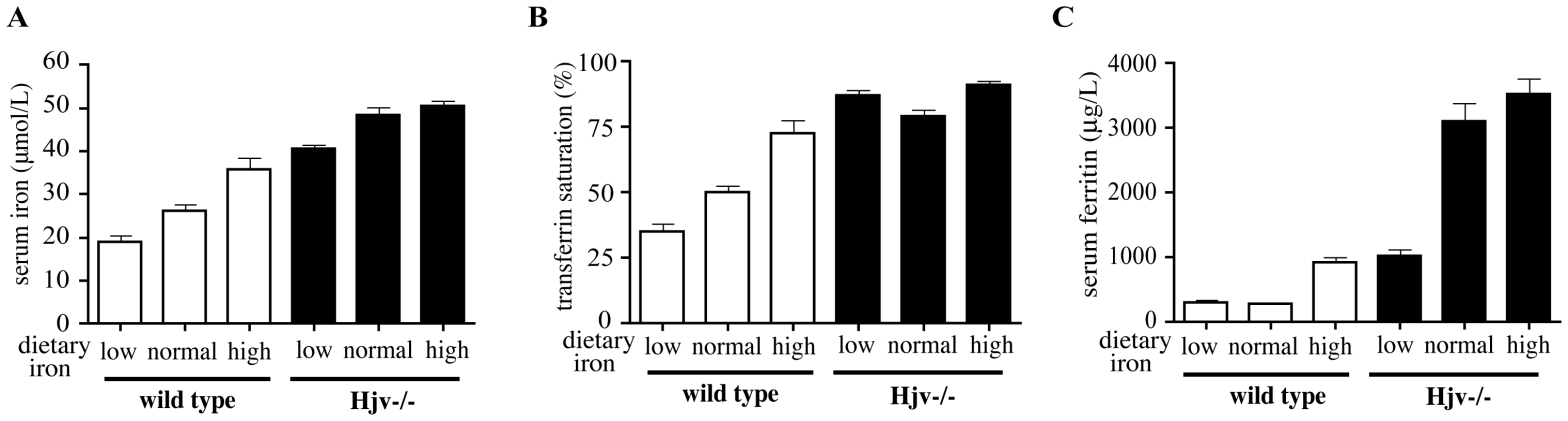
Hjv−/− mice exhibit elevated serum iron indices independently of dietary iron intake. Ten-week old male Hjv−/− and wild type mice (n = 10 for each group) in C57BL/6 background were placed on diets with variable iron content (low: 75–100 ppm; normal: 225 ppm; high: 225 ppm plus 2% carbonyl iron). After four weeks the animals were sacrificed and sera were analyzed for iron (A), transferrin saturation (B), and ferritin (C). Data are presented as the mean ± SEM. Statistical analysis is provided in Table S1.

Hjv−/− mice backcrossed to the C57BL/6 background maintained the iron overload phenotype (Table 2). Nevertheless, they accumulated less iron compared to 129S6/SvEvTac counterparts, consistently with C57BL/6 being a “low-iron” strain [18], [19]. C57BL/6 Hjv−/− mice, as well as isogenic wild type controls were subjected to dietary iron manipulations for four weeks by receiving a chow with moderately low, normal or excessive iron content. We opted to exclude a completely iron-deficient diet that may cause anemia (at least in wild type animals), to avoid potential confounding effects. The Hjv−/− mice exhibited elevated serum iron indexes (iron, Tf saturation and ferritin) under all dietary regimens, while, as expected, in wild type controls the respective values fluctuated according to dietary iron intake (Fig. 1; detailed statistical analysis of the data is provided in Table S1). Similar results were obtained with Hjv−/− and wild type mice in 129S6/SvEvTac background (Fig. S1). These data suggest that Hjv−/− mice are unable to appropriately adjust serum iron to physiological levels mirroring dietary iron supply.

**Table 2:** Serum and liver iron indices in wild type and Hjv−/− mice of 129S6/SvEvTac or C57BL/6 genetic background (n = 10 male C57BL/6 mice for each genotype; n = 5 male 129S6/SvEvTac mice for each genotype).

Genotype	wild type	wild type	Hjv−/−	Hjv−/−
**Strain**	129S6/SvEvTac	C57BL/6	129S6/SvEvTac	C57BL/6
**Serum iron (µmol/L)**	34.40±2.015	26.20±1.597**	56.80±3.734	48.50±1.682*
**Tf saturation (%)**	54.60±4.226	50.10±2.755	96.50±0.2887	79.14±2.219***
**Liver iron (µg/g dry tissue)**	652.1±68.31	255.6±11.01***	7340±776.0	6070±411.3

p<0.05;

p<0.01;

p<0.001 vs 129S6/SvEvTac mice of the same genotype (Student’s t test).

All differences among wild type and Hjv−/− mice of the same strain are statistically significant (p values not shown).

**Figure 2:**
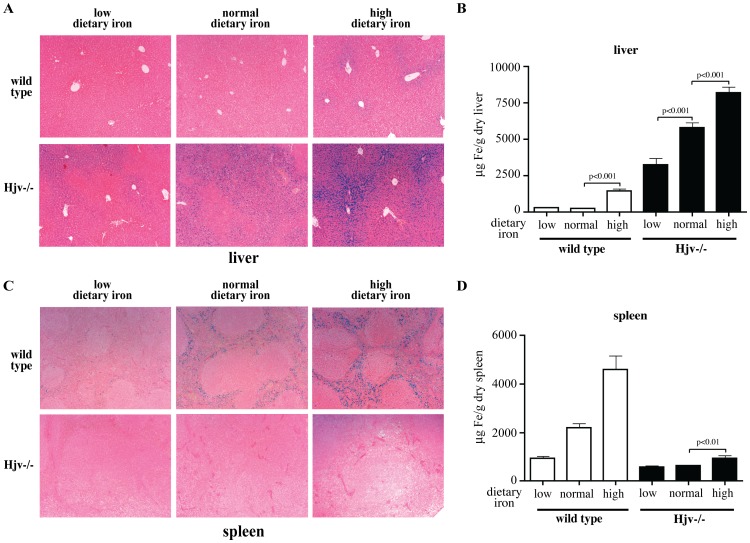
Effects of dietary iron manipulations on hepatic and splenic iron content. Livers and spleens from the Hjv−/− and wild type mice described in Fig. 1 were used for histological detection of iron by staining with Perls’ Prussian blue, and for tissue iron quantification by the ferrozine assay. (A) Visualization of ferric deposits in representative liver sections (magnification: 10×). (B) Quantification of non-heme hepatic iron. (C) Visualization of ferric deposits in representative spleen sections (original magnification: 10×). (D) Quantification of non-heme splenic iron. Data in (B) and (D) are presented as the mean ± SEM. The p values were calculated by using one-way ANOVA with Bonferroni post-test correction. Detailed statistical analysis is provided in Table S1.

Staining with Perls’ Prussian blue revealed the presence of ferric deposits in liver sections of all Hjv−/− mice, the intensity of which correlates with the dietary iron intake (Fig. 2A). By contrast, only livers of wild type mice fed with high-iron diet manifested histologically detectable iron. Tissue iron quantification corroborated these findings and confirmed the significant (p<0.01) diet-dependent increase of hepatic iron content in Hjv−/− (and wild type) mice (Figs. 2B and S2). Furthermore, this result demonstrates that the moderately iron-poor diet did not reduce the liver iron stores in wild type animals. Hjv−/− mice fed with this diet manifested higher hepatic iron levels than wild type counterparts fed with carbonyl iron, suggesting that iron overload had already developed by the time the diets were switched. Livers (and spleens) of Hjv−/− or wild type mice mice did not exhibit any histological signs of inflammation (Fig. S3).

The absence of ferric deposits in spleens of Hjv−/− mice (Fig. 2C) is consistent with their known defect in retaining iron due to accumulation of ferroportin as a result of hepcidin insufficiency [10], [11]. We noticed very faint iron staining in spleen sections from Hjv−/− animals on high-iron diet (2C, right). Quantification uncovered a ∼25% increase (p<0.01) in the amount of splenic iron in these mice compared to counterparts on normal diet (Fig. 2D). Thus, Hjv−/− mice respond to a dietary iron challenge by partially retaining splenic iron, albeit at pathologically low levels. As expected, the splenic iron content of wild type mice correlated well with dietary iron supply.

### Hjv−/− Mice Maintain a Limited Capacity to Regulate Hepcidin mRNA Expression by Dietary Iron

**Figure 3:**
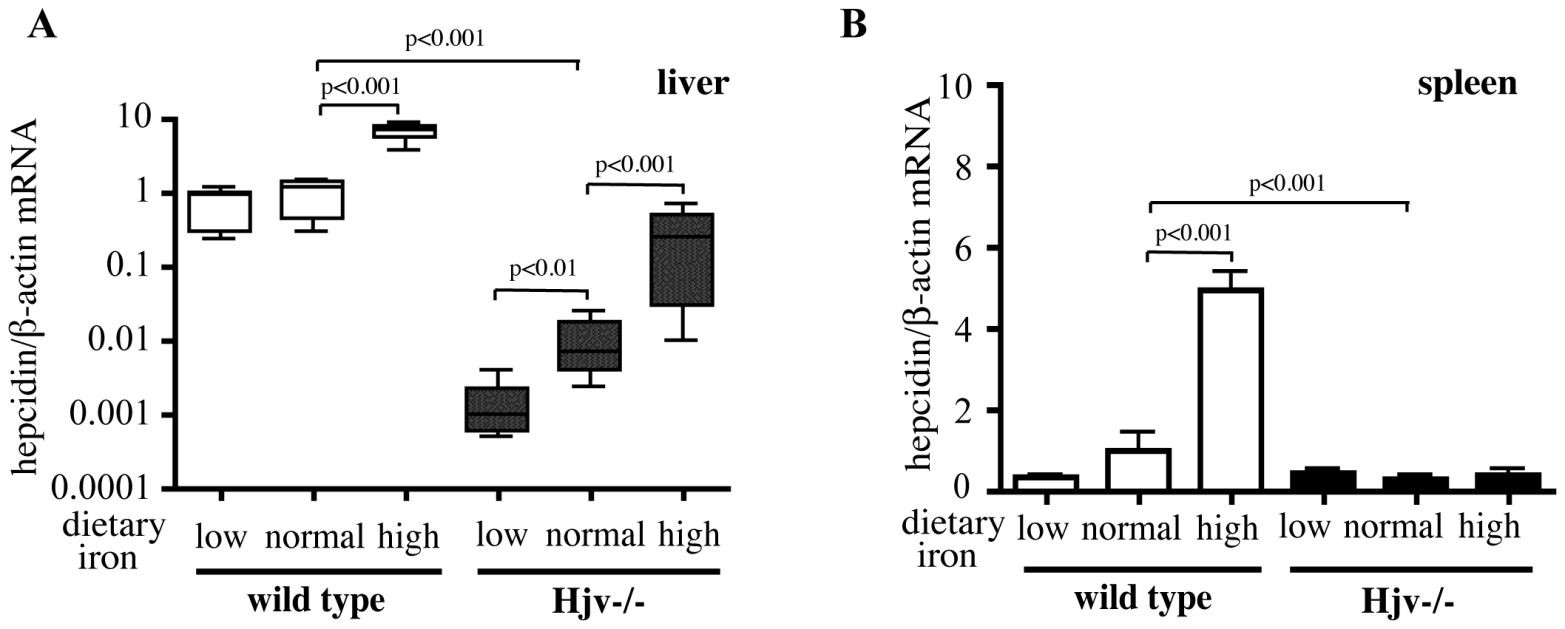
Residual iron-dependent regulation of hepatic hepcidin mRNA expression in Hjv−/− mice. RNA was extracted from tissues of the Hjv−/− and wild type mice described in Fig. 1 and used for qPCR analysis. (A) Expression of hepatic hepcidin mRNA. (B) Expression of splenic hepcidin mRNA. Note that absolute hepcidin mRNA levels in the spleen are >100 times lower than in the liver. Data are presented as the mean ± SEM. The p values were calculated by using one-way ANOVA with Bonferroni post-test correction. Detailed statistical analysis is provided in Table S1.

As expected, Hjv−/− mice expressed ∼100-fold (p<0.001) lower hepcidin mRNA levels in their livers compared to wild type controls on normal diet (Fig. 3A). Moreover, wild type animals manifested 8-fold increased hepcidin mRNA following dietary iron loading (p<0.001 vs normal diet). Surprisingly, Hjv−/− mice upregulated hepcidin expression in response to the iron-enriched diet. In quantitative terms, the iron-mediated induction of hepcidin mRNA in Hjv−/− mice was even more profound compared to wild type counterparts (∼30-fold vs normal diet, p<0.001), yet hepcidin mRNA levels remained inappropriately low. It should be noted that Hjv−/− mice on low-iron diet substantially suppressed hepcidin expression (7-fold, p<0.05 vs normal diet), while no analogous response was observed among wild type animals, in concordance with their comparable hepatic iron content. Similar results were obtained with 129S6/SvEvTac Hjv−/− mice (Fig. S4). Hence, the pathway for sensing body iron levels is largely conserved in Hjv−/− mice, while the absence of Hjv compromises the hepcidin response only quantitatively. Along these lines, the regulation of hepcidin expression by dietary iron in wild type animals was not associated with significant alterations in liver Hjv mRNA (Fig. S5).

Hepcidin mRNA was also detectable in spleens of wild type mice but its levels were negligible (>100 times lower than the liver). In Hjv−/− mice, splenic hepcidin expression was significantly (p<0.01) suppressed compared to wild type, indicating a role for Hjv in extrahepatic hepcidin regulation (Fig. 3B). Interestingly, iron accumulation promoted a ∼5-fold (p<0.001) induction of splenic hepcidin mRNA in wild type mice. Thus far, extrahepatic hepcidin production in macrophages was shown to be inducible only by inflammatory stimuli [20].

### Hjv−/− Mice Exhibit Quantitative Defects in Iron-mediated Smad Signaling Downstream of BMP6

**Figure 4:**
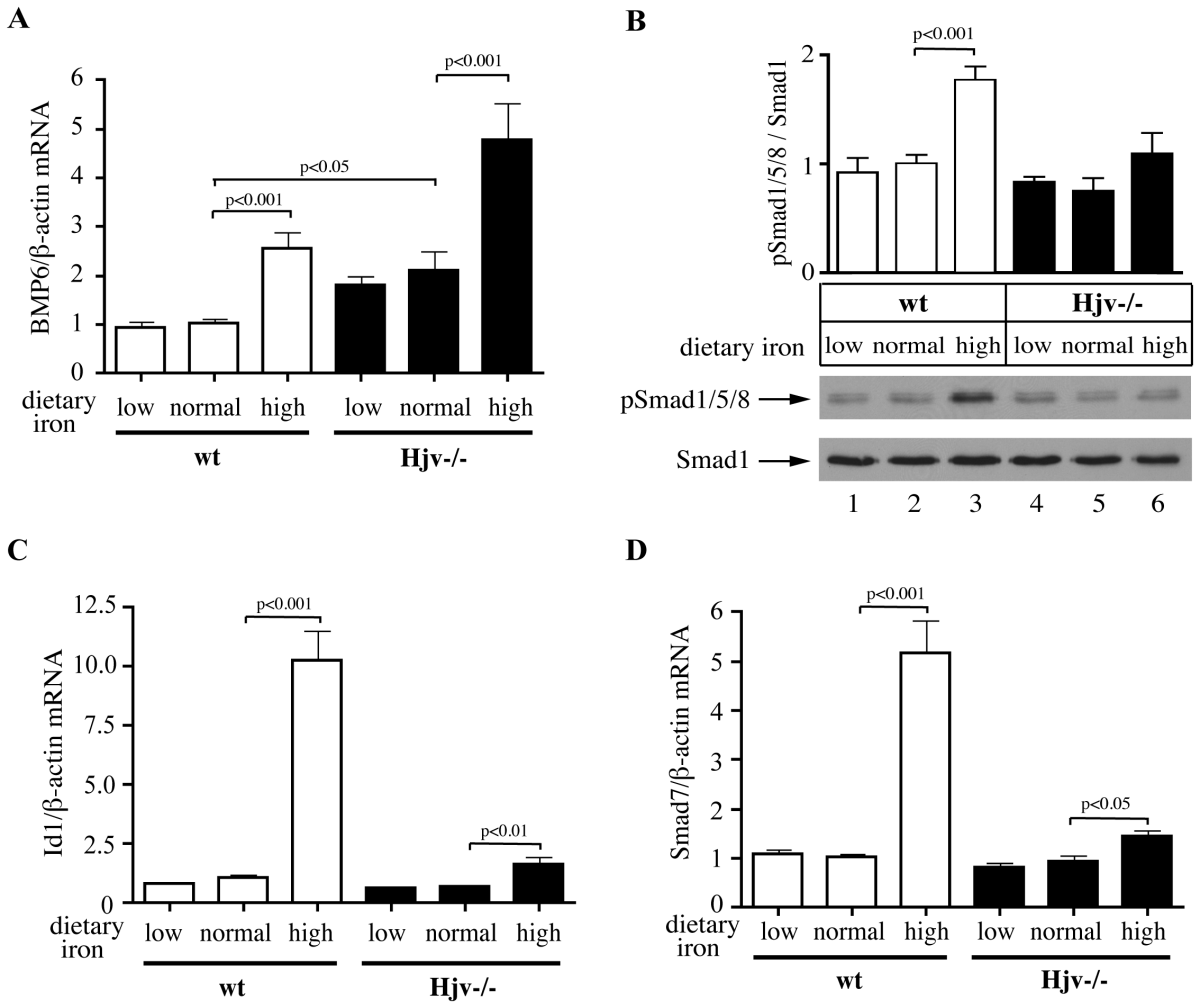
Iron-loaded Hjv−/− mice mount an appropriate BMP6 mRNA response but exhibit defective Smad signaling. RNA and protein lysates were prepared from livers of Hjv−/− and wild type mice described in Fig. 1 and used for qPCR and Western blotting, respectively. (A) Expression of Bmp6 mRNA. (B) Analysis of Smad1/5/8 phosphorylation with a phospho-specific pSmad1/5/8 antibody; a representative Western blot with samples from each group (n = 1) is shown at the bottom. Blotting with an antibody against total Smad1 serves as loading control. Densitometric quantification of all Smad1/5/8 phosphorylation data (corresponding to n = 9 mice for each group), normalized to total Smad1, is shown on top. (C and D) Expression of Id1 and Smad7 mRNA. Data are presented as the mean ± SEM. The p values were calculated by using one-way ANOVA with Bonferroni post-test correction. Detailed statistical analysis is provided in Table S1.

Hjv−/− mice displayed a 2.1-fold (p<0.05) increase in hepatic BMP6 mRNA vs isogenic wild type controls (Fig. 4A), in agreement with previous findings [13], [21], [22]. BMP6 mRNA levels were further induced by dietary iron loading in Hjv−/− mice (2.2-fold, p<0.001 vs normal diet), and were also upregulated in iron-loaded wild type controls (2.6-fold, p<0.001 vs normal diet).

The switch from normal to high-iron diet triggered robust phosphorylation of Smad1/5/8 (1.8-fold increase, p<0.001) in wild type mouse livers, but this response was blunted in Hjv−/− animals (Fig. 4B). Furthermore, under these conditions wild type mice mounted a strong induction in the mRNAs encoding Id1 (10-fold, p<0.001) and Smad7 (5.1-fold, p<0.001), which are targets of the BMP/Smad signaling pathway [23] (Figs. 4C and D). This effect was diminished in Hjv−/− mice; nevertheless, the animals displayed statistically significant residual iron-dependent stimulation of both Id1 (1.7-fold, p<0.01) and Smad7 (1.5-fold, p<0.05) mRNAs. Together, these results indicate that the lack of Hjv attenuates but does not abolish iron-dependent signaling via the BMP/Smad pathway.

## Discussion

We show that Hjv−/− mice are capable of sensing alterations in dietary iron supply and responding to them via the hepcidin pathway. However, iron-dependent induction of hepcidin is dramatically attenuated in these animals (Fig. 3) and does not suffice to mitigate further iron absorption, which contributes to deregulation of iron homeostasis and leads to iron overload. The statistically significant reduction of hepcidin mRNA content in Hjv−/− mice fed a low iron diet (Fig. 3A) suggests that Hjv is not required for negative iron-dependent regulation of the hepcidin pathway. This finding is in line with the capacity of Hjv−/− mice to decrease hepcidin expression following phlebotomy [24].

The ablation of Hjv is associated with a quantitative defect in Smad signaling. The preservation of the BMP6 mRNA induction by iron in livers of Hjv−/− mice (Fig. 4A) suggests that the signaling defect is localized downstream of BMP6, in agreement with other relevant findings [13], [21], [22]. Considering that Hjv operates biochemically as BMP co-receptor [14] our data suggest that Hjv is essential for enhancing and amplifying iron-dependent BMP6/Smad signaling to liver hepcidin. Hence, Hjv functions as an enhancer and not a dietary iron sensor, as previously postulated [10]. Niederkofler et al reached an opposite conclusion, based on experiments where hepcidin mRNA was undetectable by Northern blotting in liver samples from Hjv−/− mice that were previously injected with iron dextran [10]. By employing a more sensitive detection method (qPCR), we show here that the expression of hepcidin mRNA in Hjv−/− mice fluctuates in response to dietary iron intake, analogously to wild type animals (Fig. 3A). Notably, the magnitude of iron-dependent hepcidin induction was even higher in Hjv−/− mice compared to wild type controls switched from a normal to a high-iron diet. Nevertheless, hepcidin mRNA expression was sustained at exceedingly low levels relative to the iron load of these animals. Consistently with the function of Hjv as an enhancer and not a sensor, Hjv mRNA (Fig. S5) and protein [25] levels remained unaltered following switch of the mice from normal to high iron diet, indicating that its activity is not limiting for BMP6/Smad signaling.

Hepcidin responds to increased plasma or hepatic iron by discrete pathways [22], [26]. Dietary iron loading induced hepcidin mRNA expression without significantly affecting the already pathologically high plasma iron levels in Hjv−/− mice (Figs. 1, 3A, S1 and S4). This finding uncouples the iron-dependent induction of hepcidin from an increase in Tf saturation and suggest that hepatic iron provides the sole signal for hepcidin upregulation in these animals. This is reflected in the induction of BMP6 mRNA, which, however, does not suffice to promote significant Smad1/5/8 phosphorylation and thereby activate the BMP/Smad signaling cascade. We speculate that the limited iron-dependent upregulation of hepcidin mRNA that is observed in Hjv−/− mice is caused by residual Smad signaling activity, which is below the detection limit of the Smad1/5/8 phosphorylation assay. An iron-independent contribution of the inflammatory pathway, secondary to iron accumulation, is unlikely, considering the absence of histologically detectable inflammation in livers of Hjv−/− mice on high iron diet (Fig. S3).

The data reported here uncover the capacity of Hjv−/− mice to mount homeostatic hepcidin responses (positive or negative) to altered dietary iron supply despite the lack of Hjv. Nevertheless, without Hjv the iron-dependent induction of hepcidin is merely residual and does not suffice to prevent iron overload. Our findings corroborate conclusions of Ramos et al that Hjv is partially redundant for hepcidin upregulation by chronic dietary iron loading [22]. Nevertheless, they do not exclude a possible role of Hjv as sensor of transient increases in plasma iron. Evidence for this was provided by the complete failure of phlebotomized Hjv−/− mice with diminished plasma iron levels to upregulate hepcidin following an acute iron challenge [22]. Experiments are underway to explore the pathophysiological significance of downstream responses that are presumably linked to residual iron-dependent regulation of hepcidin, such as the relative retention of splenic iron by Hjv−/− mice on high iron diet.

## Supporting Information

Figure S1 **129S6/SvEvTac Hjv−/− mice exhibit elevated serum iron levels and fully saturated transferrin, independently of dietary iron intake.** Eight-week old Hjv−/− and wild type mice (3 male and 3 female for each group) in 129S6/SvEvTac background were placed on diets with variable iron content (low: 75–100 ppm; normal: 225 ppm; high: 225 ppm plus 2% carbonyl iron). After four weeks the animals were sacrificed and sera were analyzed for iron (A) and transferrin saturation (B). Data are presented as the mean ± SEM. Statistical analysis is provided in Table S1.
(TIF)

Figure S2 **Effects of dietary iron manipulations on hepatic iron content.** Livers from the 129S6/SvEvTac Hjv−/− and wild type mice described in Fig. S1 were isolated and used for quantification of non-heme iron by the ferrozine assay. Data are presented as the mean ± SEM. The p values were calculated by using one-way ANOVA with Bonferroni post-test correction. Detailed statistical analysis is provided in Table S1.
(TIF)

Figure S3 **Dietary iron overload for 4 weeks does not promote tissue inflammation.** H&E staining of liver (A) and spleen (B) sections of the Hjv−/− and wild type mice described in Fig. 1 (original magnification: 40×).
(TIF)

Figure S4 **Residual iron-dependent regulation of hepcidin mRNA expression in 129S6/SvEvTac Hjv−/− mice.** Liver RNA from the 129S6/SvEvTac Hjv−/− and wild type mice described in Fig. S1 was used for assessment of hepcidin mRNA by qPCR. Results represent fold changes compared to wild type mouse samples, from mice fed a normal diet. Data are presented as the mean ± SEM. The p values were calculated by using one-way ANOVA with Bonferroni post-test correction. Detailed statistical analysis is provided in Table S1.
(TIF)

Figure S5 **Liver Hjv mRNA expression does not respond to dietary iron manipulations.** RNA was extracted from the liver of Hjv−/− and wild type mice described in Fig. 1 and analyzed for Hjv mRNA expression by qPCR. Results represent fold changes compared to wild type mouse samples, from mice fed a normal diet. Data are presented as the mean ± SEM. Statistical analysis is provided in Table S1.
(TIF)

Table S1 **Detailed statistical analysis of data shown in** **Figures 1** **–** **4** **and S1–S5.** Differences across the various groups were evaluated by one-way ANOVA with Bonferroni post-test correction.
(XLSX)
