# Supplementary figures and images for: Iron-Dependent Regulation of Hepcidin in Hjv−/− Mice: Evidence That Hemojuvelin Is Dispensable for Sensing Body Iron Levels
Source: PLoS One. 2014 Jan 7;9(1):e85530. doi: 10.1371/journal.pone.0085530 (PMC3883712; doi:10.1371/journal.pone.0085530)

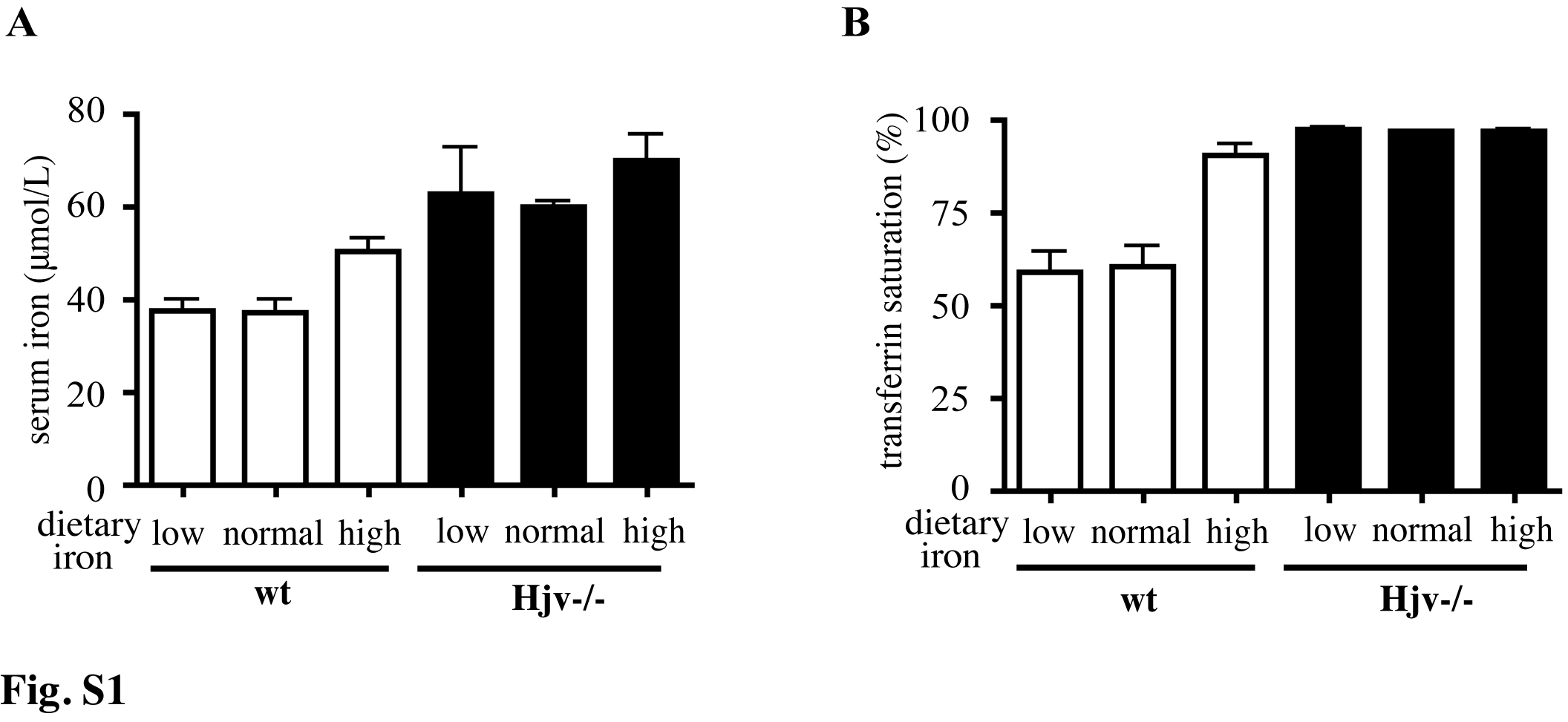

Supplement: Figure S1 — 129S6/SvEvTac Hjv−/− mice exhibit elevated serum iron levels and fully saturated transferrin, independently of dietary iron intake. Eight-week old Hjv−/− and wild type mice (3 male and 3 female for each group) in 129S6/SvEvTac background were placed on diets with variable iron content (low: 75–100 ppm; normal: 225 ppm; high: 225 ppm plus 2% carbonyl iron). After four weeks the animals were sacrificed and sera were analyzed for iron (A) and transferrin saturation (B). Data are presented as the mean ± SEM. Statistical analysis is provided in Table S1. (TIF) [file pone.0085530.s001.tif]

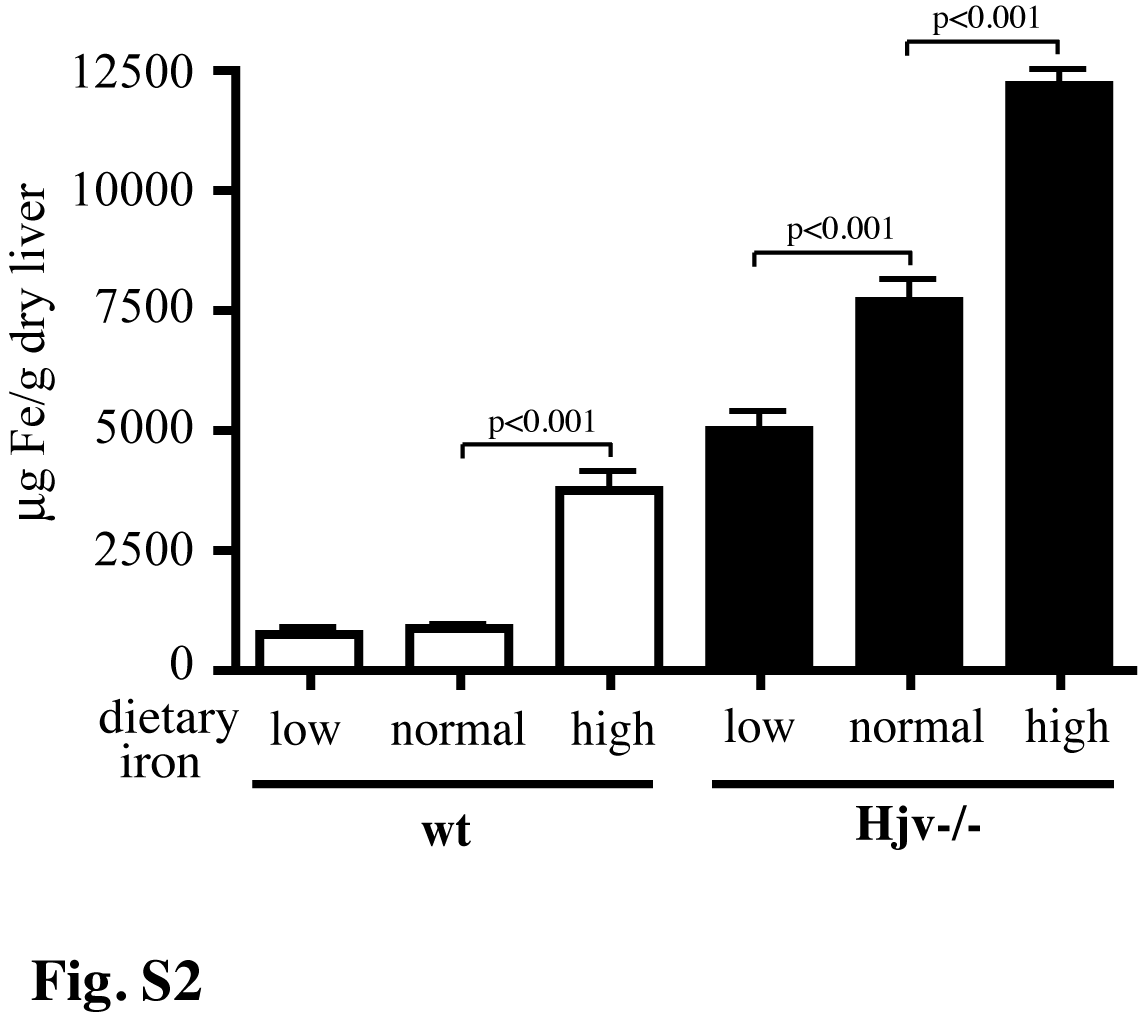

Supplement: Figure S2 — Effects of dietary iron manipulations on hepatic iron content. Livers from the 129S6/SvEvTac Hjv−/− and wild type mice described in Fig. S1 were isolated and used for quantification of non-heme iron by the ferrozine assay. Data are presented as the mean ± SEM. The p values were calculated by using one-way ANOVA with Bonferroni post-test correction. Detailed statistical analysis is provided in Table S1. (TIF) [file pone.0085530.s002.tif]

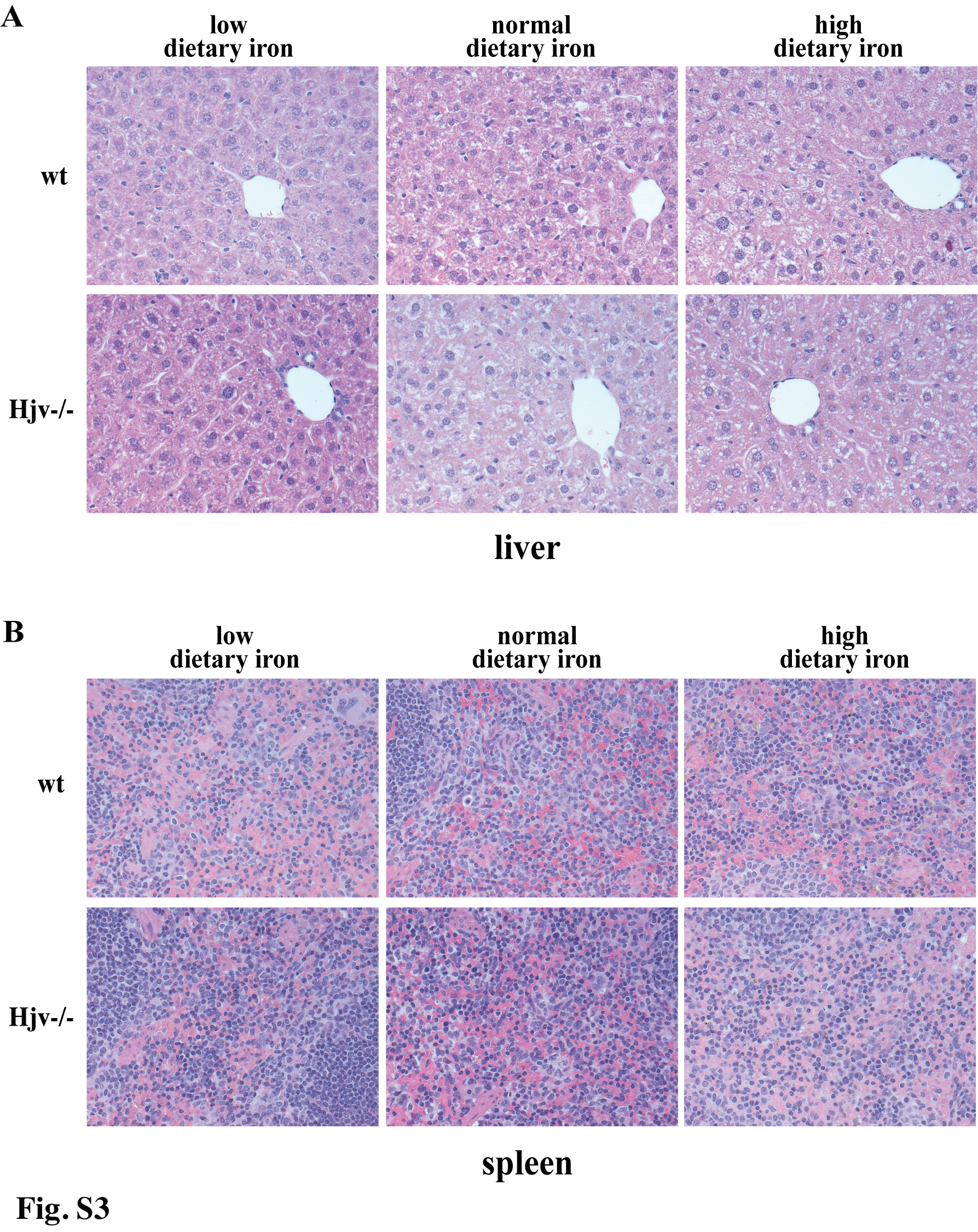

Supplement: Figure S3 — Dietary iron overload for 4 weeks does not promote tissue inflammation. H&E staining of liver (A) and spleen (B) sections of the Hjv−/− and wild type mice described in Fig. 1 (original magnification: 40×). (TIF) [file pone.0085530.s003.tif]

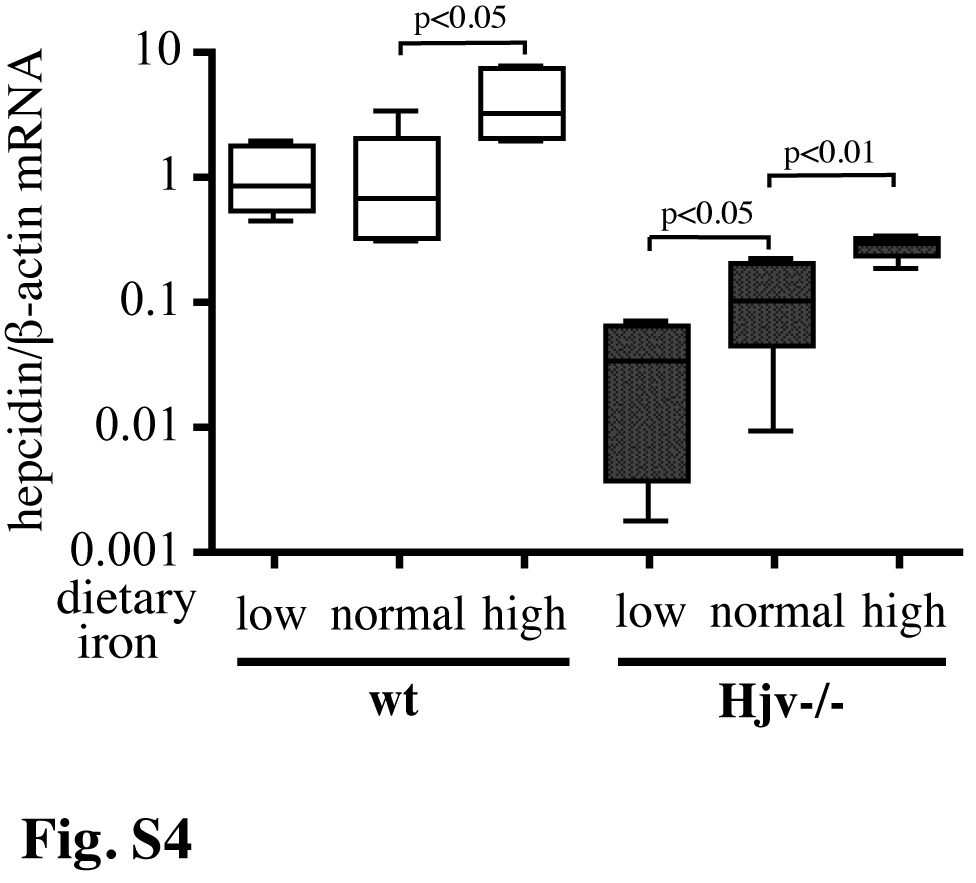

Supplement: Figure S4 — Residual iron-dependent regulation of hepcidin mRNA expression in 129S6/SvEvTac Hjv−/− mice. Liver RNA from the 129S6/SvEvTac Hjv−/− and wild type mice described in Fig. S1 was used for assessment of hepcidin mRNA by qPCR. Results represent fold changes compared to wild type mouse samples, from mice fed a normal diet. Data are presented as the mean ± SEM. The p values were calculated by using one-way ANOVA with Bonferroni post-test correction. Detailed statistical analysis is provided in Table S1. (TIF) [file pone.0085530.s004.tif]

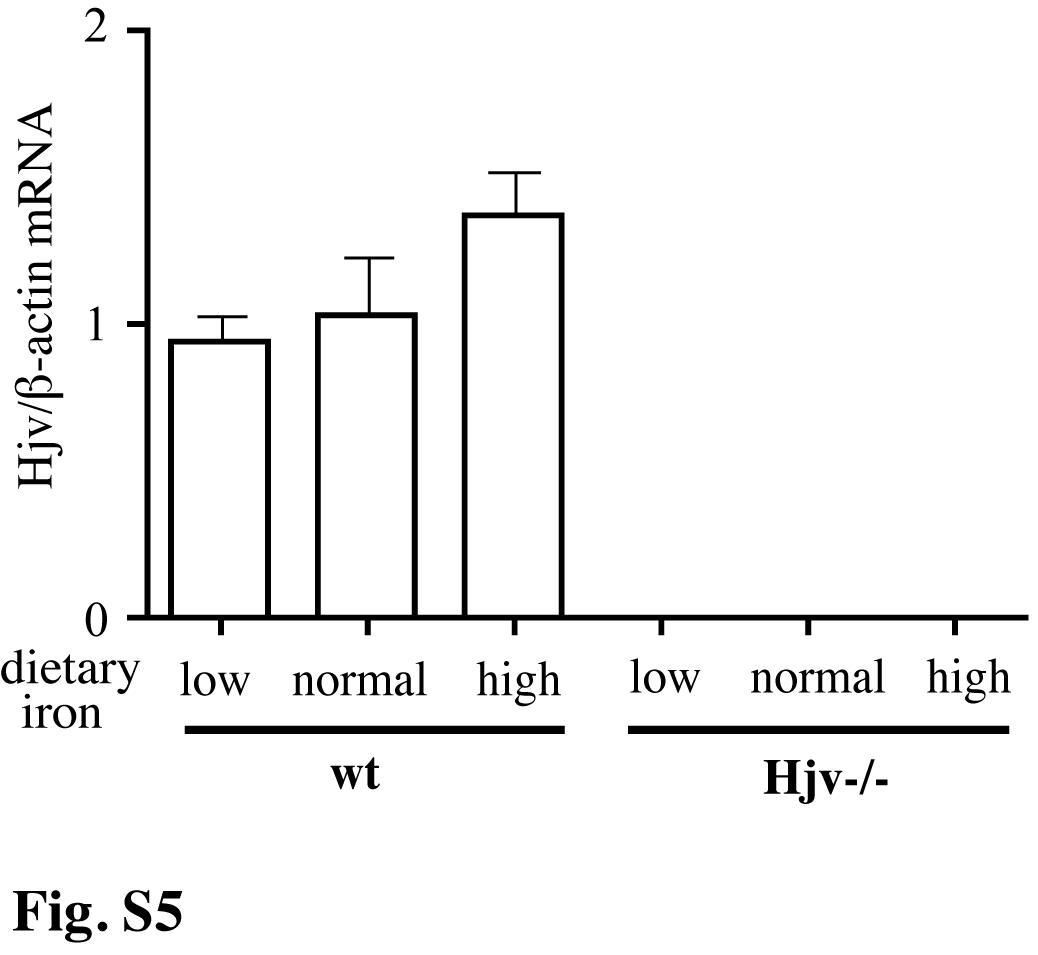

Supplement: Figure S5 — Liver Hjv mRNA expression does not respond to dietary iron manipulations. RNA was extracted from the liver of Hjv−/− and wild type mice described in Fig. 1 and analyzed for Hjv mRNA expression by qPCR. Results represent fold changes compared to wild type mouse samples, from mice fed a normal diet. Data are presented as the mean ± SEM. Statistical analysis is provided in Table S1. (TIF) [file pone.0085530.s005.tif]
